# Metastasis of mucinous breast carcinoma to the lower alveolus as initial presentation: a diagnostic dilemma

**DOI:** 10.4322/acr.2021.407

**Published:** 2022-11-03

**Authors:** Priyadarshini Guha, Zachariah Chowdhury

**Affiliations:** 1 Superspeciality Cancer Institute, Department of Pathology, Lucknow, Uttar Pradesh, India; 2 Mahamana Pandit Madanmohan Malaviya Cancer Centre & Homi Bhaba Cancer Hospital, Department of Pathology, Varanasi, Uttar Pradesh, India

**Keywords:** Adenocarcinoma, Mucinous, Breast, GATA3 Transcription Factor, Gingival, Jaw

## Abstract

Metastases in the oral cavity are rare and comprise approximately 1% of all oral malignancies. They usually involve the jaws but may also be found in the soft tissues and salivary glands. Women's most common metastatic malignancies are from primary breast cancers. However, metastasis of mucinous breast carcinoma to the lower alveolus mimicking an aggressive primary malignancy as the initial presentation is exceptionally uncommon. We describe the case of a 66-year-old lady with an ulceroproliferative growth in the right lower alveolus. The lesion eroded the mandible and involved the adjacent soft tissues with no prior history of lesion anywhere else. The lesion clinically mimicked a squamous cell carcinoma and masqueraded as a salivary gland mucinous adenocarcinoma on histopathology. The possibility of a metastatic lesion from the breast rather than a primary of the alveolus was also entertained, aided by the immunohistochemical findings of positivity of the tumor cells for GATA3. A positron emission tomography (PET) scan was undertaken to ascertain the primary site. It detected a hypermetabolic lesion in the left breast, which biopsy revealed mucinous breast carcinoma on histopathological evaluation. Metastasis of breast mucinous carcinoma by the hematogenous route is extremely rare; very few cases have been reported. This case illustrates the diagnostic challenges such a lesion can pose to the surgeon and the pathologist. In the advent of such lesions being the initial clinical presentation, a vigilant clinicopathological and radiological assessment is essential to detect the primary.

## INTRODUCTION

Mucinous breast cancer is a rare neoplasm of the breast, accounting for approximately 2% of all breast carcinomas.^1^ It is a type of invasive breast carcinoma characterized by clusters of epithelial tumor cells floating in pools of extracellular mucin.^2^ The initial presentation of such a neoplasm as a metastatic lesion in the lower alveolus is sporadic and can present as a diagnostic peril. No prior history of any primary lesion elsewhere and negative findings on clinical examination confounds the diagnosis. The authors report this case to highlight and emphasize the role of histopathology, pathological awareness, and immunohistochemistry in resolving such situations and guide subsequent investigations to detect the primary.

## CASE REPORT

**Figure 1:**
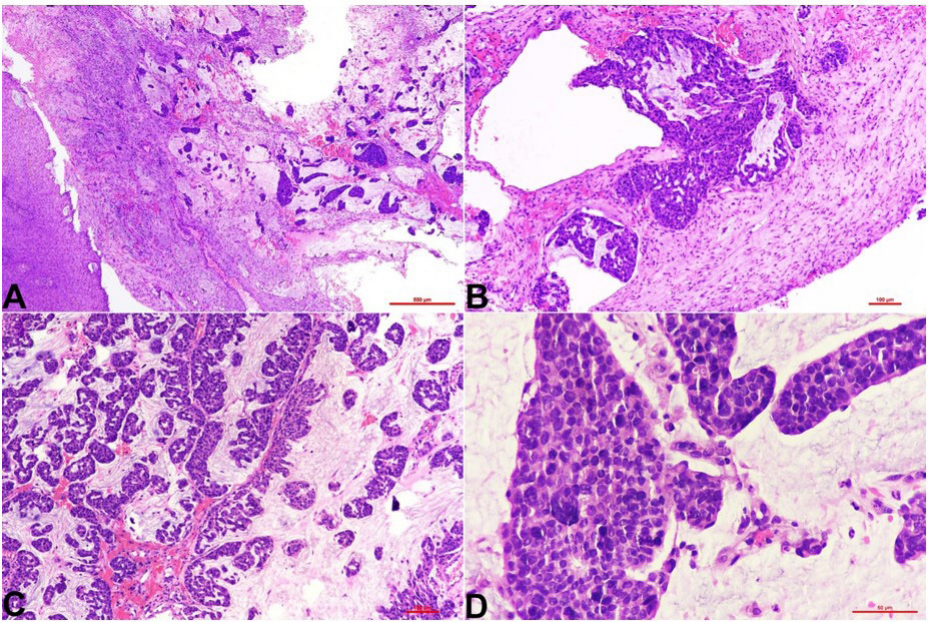
Photomicrographs of the histopathology of gingival soft tissue exhibiting hyperplastic uninvolved squamous mucosa with the subepithelial infiltration by tumor cells arranged in papillae and clusters embedded in pools of mucin; H&E stain, **A -** 4X, **B** and **C -** 10X, and **D -** 40X.

A 66-year-old lady presented to our institute with chief complaints of non-healing ulcer, pain, and swelling in the right lower alveolus for one month. An ulceroproliferative growth measuring approximately 5cm x 3cm was seen on the right lower alveolus, extending from the right first premolar to the retromolar trigone. The lesion was firm in consistency, indurated, and not associated with bleeding. No palpable lymph nodes were identified. A contrast-enhanced computed tomography (CT) scan of the head and neck showed a destructive lytic lesion involving the ramus and the coronoid process of the mandible with soft tissue component involving masseter, masticator space, and medial pterygoid along with compressed D3 vertebrae and no significant lymph nodes. Clinically suspecting it to be a squamous cell carcinoma eroding the mandible, an incisional biopsy of the lesion was performed. The histopathological examination (HPE) revealed a tissue partly lined by hyperplastic mature stratified squamous epithelium, with the underlying subepithelium exhibiting extracellular pools of mucin admixed with neoplastic cells in cohesive syncytial aggregates and glandular formations. The cells were round to oval and displayed regular nuclei and a moderate amount of cytoplasm. Marked nuclear pleomorphism was not identified (Figure 1).

**Figure 2:**
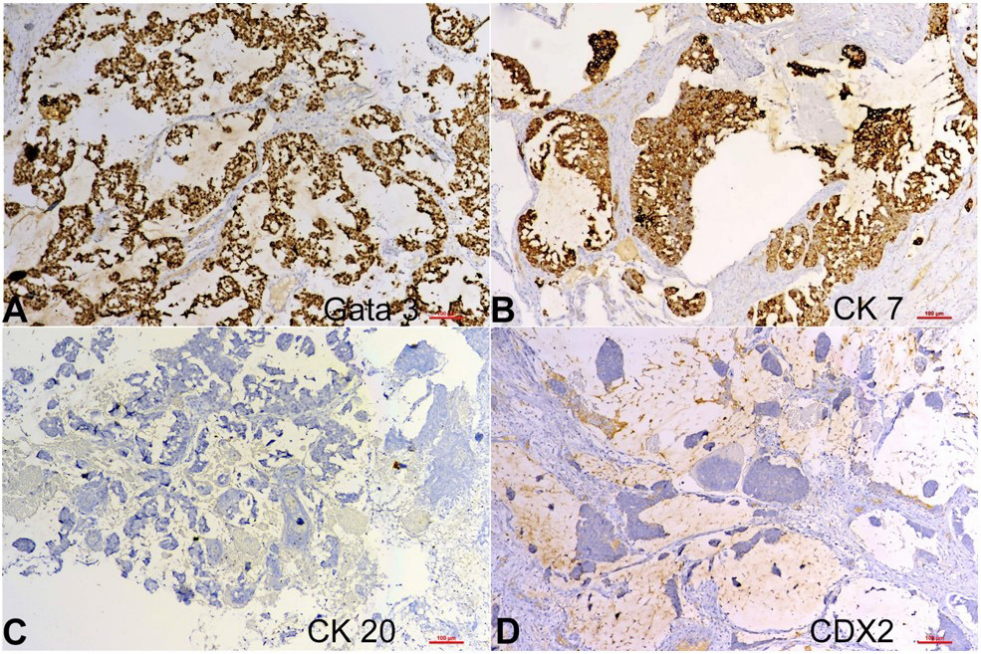
Photomicrographs of the tumor in Figure 1 displaying immunohistochemical positivity for **A -** GATA3 and **B -** CK7 and negativity for **C -** CK20 and **D -** CDX2; (**A**, **B**, **C**, **D**) 10X.

The initial impression on the Hematoxylin and Eosin (H&E) stained slides was a mucinous adenocarcinoma. The IHC disclosed the neoplastic cells to be positive for CK7 and GATA3 and negative for CK20, CDX2, and TTF1 (Figure 2). Based on the histopathological findings and the immunophenotype, two possibilities were considered; the first was a primary mucinous adenocarcinoma of the minor salivary glands, and the second was a metastatic adenocarcinoma, probably of breast origin. A guiding remark in the HPE report mentioned a thorough investigation of the primary site.

**Figure 3:**
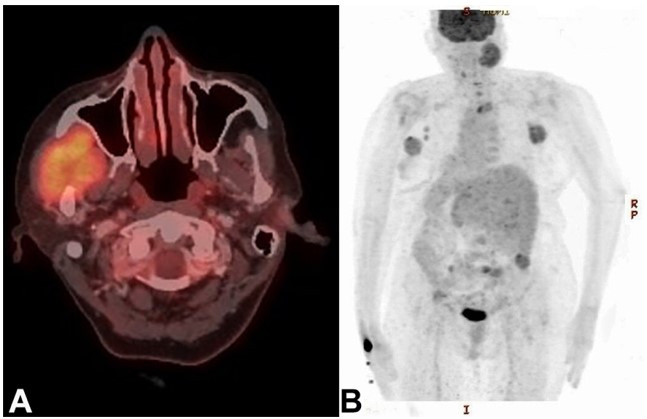
Positron emission tomography (PET) scan demonstrating: **A -** enhancing destructive lytic lesion involving ramus and coronoid process of the right mandible and adjacent soft tissues, and **B -** hypermetabolic left axillary nodes and skeletal metastases involving lamina of C7, D1, and D3 vertebra, left acetabulum, bilateral iliac bones, sacrum, and right scapula with associated soft tissue components.

The patient was then subjected to Fluorodeoxyglucose (FDG) Positron Emission Tomography (PET) scan for possible primary and initial staging. Apart from an FDG avid enhancing destructive lytic lesion involving ramus and coronoid process of the right mandible with associated soft tissue component involving right masticator space, right masseter, medial pterygoid muscle, measuring 50 x 40 mm with SUVmax 7.78, the imaging modality also divulged an FDG avid heterogeneously enhancing mass in the outer quadrant of the left breast, measuring 45 x 40 mm with SUVmax 7.06. Hypermetabolic left axillary nodes and skeletal metastases involving lamina of C7, D1, and D3 vertebra, left acetabulum, bilateral iliac bones, sacrum, and right scapula with associated soft tissue components were also identified (Figure 3).

**Figure 4:**
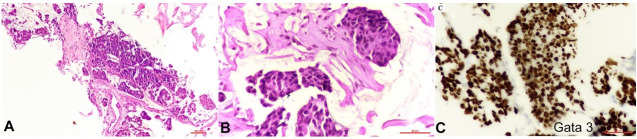
Photomicrographs of the histopathology of mucinous breast carcinoma showing **A** and **B -** tumor cells in extracellular pools of mucin (10X and 40X respectively); **B -** positive reaction for GATA3 on immunohistochemistry (40X).

Subsequently, a core biopsy from the left breast mass was performed, HPE displayed extracellular mucin (> 90%) with few invasive tumor cells correlating with a diagnosis of mucinous breast carcinoma, grade 2. The tumor cells were diffusely positive for GATA3 on IHC (Figure 4).

The biomarker profile was both Estrogen receptor (ER) and Progesterone Receptor (PR) positive, with Her2/neu negativity. Thus, the patient was confirmed to suffer from mucinous carcinoma, grade 2 of the left breast, with metastasis to the right lower alveolus and other sites. Given the widespread malignancy and unresectability of the alveolus lesion reaching above the zygoma and the retromandibular area, the treatment plan was modified to palliative chemotherapy and palliative radiotherapy to the mandible. A follow-up PET-CT scan done for evaluation of disease status after treating the patient with fulvestrant and anastrozole showed significantly reduced metabolic activity compared to the baseline parameters, exhibiting low-grade FDG uptake in the left breast mass - SUVmax 2.41 (previous SUVmax 7.06), low-grade FDG uptake in the lytic lesion of the mandible - SUVmax 3.92 (previous SUVmax 7.78) and low-grade FDG uptake in the left axillary nodes - SUVmax 2.35 (previous SUVmax 3.82).

## DISCUSSION

Pure mucinous carcinoma is an uncommon variant of breast carcinomas, characterized by clusters of generally small and uniform cells floating in large amounts of extracellular mucin. The mucinous component must constitute > 90% of the entire tumor for it to be termed a mucinous carcinoma; otherwise, the better terminology is mixed or mucinous variant of invasive breast carcinoma. It mainly occurs in older women, with the median age of presentation being 71 years. Pathogenesis of mucinous carcinoma has revealed that it is a luminal A molecular subtype.^3^ Grossly the tumor presents as a gelatinous nodule with pushing margins and viscous consistency. The size ranges from < 1 cm to > 20 cm. Histopathology has revealed mucinous carcinoma consisting of clusters of neoplastic cells floating in pools of extracellular mucin, partitioned by delicate fibrous septa containing capillary blood vessels. Individual tumor cells generally have low to intermediate nuclear grades. As per the recent WHO classification of tumors of the breast, two morphological types are discerned, type-A (hypocellular) and type-B (hypercellular).^4^ On IHC, mucinous carcinoma breast is positive for ER, PR, and Androgen Receptor in 80% of cases. Her2/neu overexpression is rare in mucinous carcinoma but is found in > 10% of mucinous carcinoma with micropapillary pattern.^5^ The tumor in the present study satisfied the criteria for mucinous carcinoma and demonstrated positivity for ER, PR, and negativity for Her2/neu.

Involvement of the oral cavity by malignant metastatic tumors has been infrequently reported, only in 1% of all oral malignancies.^6^^-^8 Soft tissues of the oral cavity are less frequently involved by metastatic malignant tumors as compared with the bone, and metastasis to the gingiva is very rare.^9^^,^^10^ Metastasis to the oral cavity from the upper respiratory and upper gastrointestinal tracts are the most common.^11^ Invasive breast carcinoma, regardless the type, is the most common type of breast malignancy reported in literature metastasizing to the gingival.^10^^,^^11^ An extensive English literature review did not find gingival metastasis of mucinous breast carcinoma. Metastasis in mucinous breast carcinoma occurs at a lower frequency than other invasive carcinoma breast types.^12^ Radiological evaluation of the gingival lesion helps assess the tumor’s benign or malignant nature, location, and extent. However, imaging criteria are unable to distinguish a primary malignant tumor from a metastasis.^13^ For accurate diagnosis the pathologist should be aware of the metastatic tumor that can mimic primary mucinous carcinoma of the salivary gland. All the criteria for the diagnosis of a metastatic tumor,^14^^,^^15^ such as histological proof of the primary malignancy, the similarity of the histological type of the metastatic tumor with the primary neoplasm, and no direct extension from the primary tumor, are fulfilled by our case. The confusing aspect, in this case, was the primary presentation as a gingival ulceroproliferative lesion. No history of any breast lesion was known nor were any symptoms/signs related to a breast lesion from the patient registered. The diagnostic dilemma was confounded in such a scenario, with a histologic picture of mucinous carcinoma. The pathologist was, however aware enough to consider metastasis from the breast as one of the differential diagnoses, and consequently, IHC was taken help of. While encountering mucinous neoplasm at unusual sites, the possibility of metastasis should not be ignored. Though GATA3 was positive on IHC, it did not conclusively rule out salivary gland malignancy, although favoring the notion of metastasis from the breast. The case was discussed with the head and neck surgeon, and the importance of further imaging investigation, such as PET-CT, was emphasized for delineation of the nature of the lesion as a metastatic/primary. Thus, the role of histopathology and the awareness of the pathologist cannot be understated.

Mucinous carcinoma otherwise has a favorable prognosis with an excellent 5-year disease-free survival.^16^ For the patients with metastatic lesions of the oral cavity, the prognosis is usually poor mainly because of the delay in detecting the lesions. The survival time for patients with oral metastatic tumors is six to seven months on average, with about 70% of patients succumbing within one year of diagnosis.^17^ In our case, the patient was treated with palliative chemotherapy and palliative radiotherapy, and a follow-up PET-CT scan was done for the disease evaluation, and evinced significantly reduced metabolic activity compared to pretreatment values.

To conclude, the authors wish to highlight the sporadic phenomenon of gingival metastasis of breast mucinous carcinoma as the initial presentation and the inherent diagnostic quandaries. It is to be remembered that an oral lesion may be the first sign of a distant malignancy. In the conservative Indian scenario, sometimes a history of breast lesion may not be forthcoming due to various non-medical reasons. In such a situation, it is pertinent that a meticulous examination is not missed by the clinician. Even in the setting of a negative prior history, pathological awareness and acumen are of paramount importance to apprehend such an unusual diagnosis, either on HPE or cytopathology. Metastasis should be in the differential diagnosis of an oral lesion if not conforming to conventional histology. It should be borne in mind that a breast primary may masquerade pathologically as a salivary gland neoplasm and take everyone for a ride. Therein lies the significance of a vigilant clinico-radio-pathological assessment.
